# From Brain Models to Robotic Embodied Cognition: How Does Biological Plausibility Inform Neuromorphic Systems?

**DOI:** 10.3390/brainsci13091316

**Published:** 2023-09-13

**Authors:** Martin Do Pham, Amedeo D’Angiulli, Maryam Mehri Dehnavi, Robin Chhabra

**Affiliations:** 1 Department of Computer Science, University of Toronto, Toronto, ON M5S 1A1, Canada; martindopham@gmail.com (M.D.P.); mmehride@cs.toronto.edu (M.M.D.); 2 Department of Neuroscience, Carleton University, Ottawa, ON K1S 5B6, Canada; amedeodangiulli@cunet.carleton.ca; 3 Department of Mechanical and Aerospace Engineering, Carleton University, Ottawa, ON K1S 5B6, Canada

**Keywords:** neuromorphics, spiking neural networks, embodied cognition, neurorobotics

## Abstract

We examine the challenging “marriage” between computational efficiency and biological plausibility—A crucial node in the domain of spiking neural networks at the intersection of neuroscience, artificial intelligence, and robotics. Through a transdisciplinary review, we retrace the historical and most recent constraining influences that these parallel fields have exerted on descriptive analysis of the brain, construction of predictive brain models, and ultimately, the embodiment of neural networks in an enacted robotic agent. We study models of Spiking Neural Networks (SNN) as the central means enabling autonomous and intelligent behaviors in biological systems. We then provide a critical comparison of the available hardware and software to emulate SNNs for investigating biological entities and their application on artificial systems. Neuromorphics is identified as a promising tool to embody SNNs in real physical systems and different neuromorphic chips are compared. The concepts required for describing SNNs are dissected and contextualized in the new no man’s land between cognitive neuroscience and artificial intelligence. Although there are recent reviews on the application of neuromorphic computing in various modules of the guidance, navigation, and control of robotic systems, the focus of this paper is more on closing the cognition loop in SNN-embodied robotics. We argue that biologically viable spiking neuronal models used for electroencephalogram signals are excellent candidates for furthering our knowledge of the explainability of SNNs. We complete our survey by reviewing different robotic modules that can benefit from neuromorphic hardware, e.g., perception (with a focus on vision), localization, and cognition. We conclude that the tradeoff between symbolic computational power and biological plausibility of hardware can be best addressed by neuromorphics, whose presence in neurorobotics provides an accountable empirical testbench for investigating synthetic and natural embodied cognition. We argue this is where both theoretical and empirical future work should converge in multidisciplinary efforts involving neuroscience, artificial intelligence, and robotics.

## 1. Introduction

Understanding how living organisms function within their surrounding environment reveals the key properties essential to designing and operating next-generation intelligent systems. We refer to a system as intelligent if it has the capability of fast adapting to the changes in its components, environment, or mission requirements. A central actor enabling organisms’ complex behavioral interactions with the environment is indeed their efficient information processing through neuronal circuitries. Although biological neurons transfer information in the form of trains of impulses or spikes, Artificial Neural Networks (ANNs) that are mostly employed in the current Artificial Intelligence (AI) practices do not necessarily admit a binary behavior. In addition to the obvious scientific curiosity of studying intelligent living organisms, the interest in the biologically plausible neuronal models, i.e., Spiking Neural Networks (SNNs) that can capture a model of organisms’ nervous systems, may be simply justified by their unparalleled energy/computational efficiency.

Inspired by the spike-based activity of biological neuronal circuitries, several neuromorphic chips, such as Intel’s Loihi chip [1], have been emerging. These pieces of hardware process information through distributed spiking activities across analog neurons, which, to some extent, trivially assume biological plausibility; hence, they can inherently leverage neuroscientific advancements in understanding neuronal dynamics to improve our computational solutions for, e.g., robotics and AI applications. Due to their unique processing characteristics, neuromorphic chips fundamentally differ from traditional von Neumann computing machines, where a large energy/time cost is incurred whenever data must be moved from memory to processor and vice versa. On the other hand, coordinating parallel and asynchronous activities of a massive SNN on a neuromorphic chip to perform an algorithm, such as a real-time AI task, is non-trivial and requires new developments of software, compilers, and simulations of dynamical systems. The main advantages of such hardware are (i) computing architectures organized closely to known biological counterparts may offer energy efficiency, (ii) well-known theories in cognitive science can be leveraged to define high-level cognition for artificial systems, and (iii) robotic systems equipped with neuromorphic technologies can establish an empirical testbench to contribute to the research on embodied cognition and to explainability of SNNs. Particularly, this arranged marriage between biological plausibility and computational efficiency perceived from studying embodied human cognition has greatly influenced the field of AI.

In any interdisciplinary research, where neural networks are used to construct entirely artificial systems or to simulate living intelligent systems, defining cognition from different disciplines’ points of view becomes a “wicked problem” [2]. For an engineer, it may be enough to include artificial neural systems capable of state-of-the-art task performance that are designed based on biological principles and are not necessarily faithful to in silico representations of neural circuitry in vivo. On the other hand, a neuroscientist may only be interested in seeking biologically-equivalent representations that are not necessarily computationally efficient. Both describe what they investigate as “cognition”, and both are indeed partially correct (we distinguish between artificial and biological cognition here) until a strong link is drawn from the in vivo observations to in silico realizations. A clear distinction can be observed when considering neuromorphic technologies, where better energy efficiency overshadows the inherent biological plausibility of what can be deemed cognition.

Connecting the dots from in vivo to in silico neural systems requires not only an account of physical substrates dynamics supporting cognition, as observed in nature, but also their replication in viable neural computing architectures to reproduce such observations. This perspective provides a platform to test purely embodied versus purely functionalist computational hypotheses of mind: Are certain classes of biologically embodied computations (e.g., sensorimotor processing for perception and locomotion) inherent features of the physical substrates? Or, is the symbolic information processing universal to the substrates? Embodied analog-hardware cognition suggests that certain features of the mind are contingent upon the physical substrates (sensing organs and actuators) in which it is embodied. That is, mental processes are the exclusive province of the brain and they are naturally dependent on and constrained by the physical characteristics of the neural system through which their operations are implemented (the so-called neural dependency). Although symbolic computationalist theories of mind may also have roots in neural activities, they hold the belief that the mind is the consequence of what neural information processing does, i.e., its functionality, not the specific hardware [3]. Without being bogged down in the endless philosophical debate around the classic mind-body problem, we can pragmatically conclude that a dialectical synthesis of these tensions suggests that a limited class of substrates is capable of performing a limited class of computations and that these classes are constrained by the biological entity and task at hand [4]. Consistent with this pragmatic approach, the interdisciplinary link between neuroscience and AI may in fact be determining these reciprocal constraints. At this critical junction, robotic experimentation of embodied cognition developed predicated on SNNs becomes the necessary test bench [5]. Notably, many outstanding questions of the field then collapse into the following: (i) Do biological neuronal models that better reproduce observed empirical data better capture cognition (i.e., intelligent behaviors), when implemented on robotic agents?, and (ii) How do the embodiment of neural networks affect the network dynamics and topology, when interacting with the robot’s physical environment?

This article examines some recent multidisciplinary developments in the use of SNNs for both natural and engineering sciences. We summarize and critically review new developments in three separate areas: (i) vision and image processing using Dynamic Vision Sensors (DVS), (ii) SNN-based analysis of the Electroencephalography (EEG), and (iii) robust spiking control systems applicable in robotic Guidance, Navigation, and Control (GNC). By connecting the dots from perception to signal processing for brain data to GNC systems for robotics, we highlight where SNNs may be used to further research goals across disciplines and how they may be constituent to a theory of embodied cognition. A survey of control based on learning-inspired spiking neural networks can be found in [6] where different learning rules are identified and classified for robotic applications. More recently, [7] exclusively reviews the application of neuromorphic computing for socially interactive robotics and [8] focuses on the implementation of neuromorphic hardware in robotics. However, comprehensive research on closing the cognition loop in neurorobotics is still missing in the literature, which is our main agenda in the current paper.

The paper is structured as follows: Section 2 provides a deep description of SNN modeling, distinguishing between descriptive models and functional models. Section 3 discusses the bottlenecks in the computational simulation and emulation of SNNs. Section 4 discusses the embodiment of SNN into cognitive robotic systems and reviews multiple perspectives that SNNs can play a crucial role in developing an intelligent cognitive robot. It also explores the consequences of the technological advancements to future brain-inspired computational cognition research in relation to simulating adaptable and reconfigurable neurorobotic systems. Section 5 summarizes the trends in the literature and the limitations of the existing technologies. Some concluding remarks are included in Section 6.

## 2. SNNs and Neuromorphic Modeling

**Figure 1:**
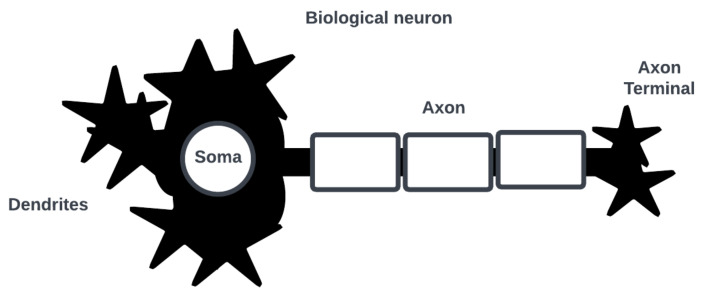
Simplified diagram of biological neuron and its main parts of interest.

In this section, we recall the basic components of SNN modeling and discuss their neuromorphic implementation [9,10]. For a more detailed review of SNNs and their applications, we refer the reader to [11]. Note that the category of bio-inspired computing is an unsettled one, and it is unclear how such systems can be designed to integrate into existing computing architectures and solutions [12]. Consider the neuron as the basic computational element of a SNN [13]. Figure 1 shows a simplified biological neuron consisting of dendrites where post-synaptic interactions with other neurons occur, a soma where an action potential (i.e., a spike in cell membrane voltage potential) is generated, and an axon with a terminal where an action potential is triggered for pre-synaptic interactions.

**Figure 2:**
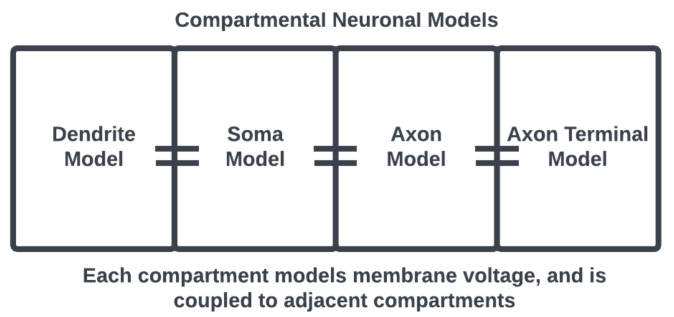
Compartmental neuronal model, the adjacency of compartments considers the physical structure of a neuron. Compartments may also be prescribed with appropriate subcellular morphology.

One way to model a spiking neuron is using compartmental models [14] that consider each of these morphological components separately, with their own sets of dynamical equations coupled together to represent the functional anatomy of a neuronal cell [15]. For example, as in Figure 2, the equations describing the membrane potential associated with the dendrites are only coupled to the soma but the equations associated with the soma are coupled to the axon as well. Thus, interactions at the subcellular level are modeled locally to represent cell anatomy.

The advantage of this model is the structural details and subcellular local effects that may be included; however, the model of a neural network becomes too complex and difficult to compute. In this sense, compartmental models may be viewed as biologically plausible but computationally unfeasible for many applications beyond investigations at the cellular level. The work of [16] implemented a multi-compartmental model on the SpiNNaker neuromorphic hardware to incorporate dendritic processing into the model of computation.

**Figure 3:**
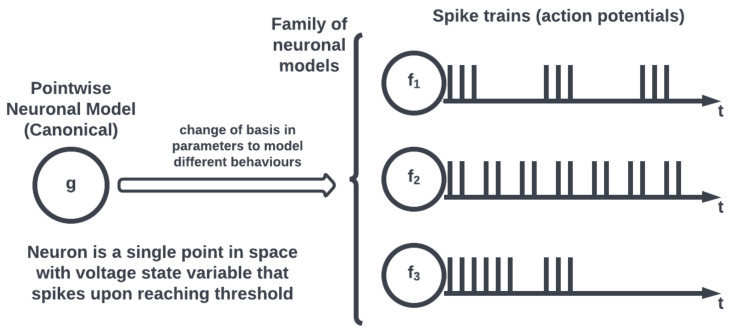
Pointwise neuronal models and canonical equations.

On the other hand, most neuromorphic hardware implements a simplified form of neuronal models, known as a pointwise model (see Figure 3), where a neuron is conceptualized as a single point. Pointwise models abstract the intracellular dynamics into a state voltage variable that records a spike when the voltage exceeds a threshold to effectively capture the action potential behavior of the neuron without having to model its structure. Therefore, unlike compartmental neuronal models that capture the spatiotemporal neuronal dynamics allowing for electrochemical process simulations, pointwise models offer computationally efficient simulations for large-scale networks neglecting spatial complexity.

The difference between compartmental and pointwise neuronal models is just one design decision when simulating neural systems, and it can be argued that the latter lacks biological plausibility. A now near-canonical example of neuronal modeling is the Hodgkin–Huxley model describing the dynamics of action potentials in neurons [17]. In the case of Hodgkin–Huxley, a continuous-time system of differential equations forming a mathematical template for neuronal modeling was derived by selecting squid as a biological specimen and analyzing its nervous system. Despite its descriptive completeness and prescriptive accuracy, this formalization is still an abstraction suffering from the representation problem of the map-territory relation [18]. Furthermore, the required computational machinery to implement the simulation of a single neuron based on the Hodgkin–Huxley model outweighs its biological completeness [19]. Generally, it is undeniable that the computational feasibility of any model inherently comes at the cost of some inevitable reduction in biological veridicality. Following Hodgkin–Huxley’s seminal work, much development has been made in capturing the dynamics of neuronal cells. The work of Izhikevich [20] showed that many observed spiking patterns could be reproduced using a small set of differential equations and that these equations were also simpler to compute while making a tradeoff in physically meaningful quantities. The eponymously named model consists of a pair of first-order differential equations on the space of the membrane potential and recovery variable of a neuron along with a simple spiking thresholding logic. Related works named these equations the canonical model approach, where the variability of spiking behaviors could be accounted for using a change of basis in the variables of the dynamical equations [21]. Accordingly, a neuronal model can be re-written in the form of the canonical system which allows for a consolidation of neuronal activity analysis under one dynamical system. The advantage of this approach is that an empirically observed spiking behavior may be modeled by a complicated and biologically detailed model or a simplified and reduced form, depending on needs and resources. The canonical approach also allows for the investigation of different kinds of spiking behavior (bursting, phasic) generated by the same neuronal dynamics under different parameter regimes, which is a step towards the standardization of neuronal models matching observed empirical data. Under this approach, dynamical systems theory bears upon our neurophysiological understanding via simulating the (biological) spiking behavior of different types of neuronal cells employing the appropriate canonical model with specific parameterizations [22]. The problem of modeling neurons’ spiking behavior then transforms into selecting the differential equation and estimating its parameters. It is from this formalized neuronal dynamics position that the GNC of neurorobotic systems is revisited in a later section. A discussion and summary table of biological plausibility and computational efficiency for different neuronal models, including Hodgkin–Huxley, Izhikevich, and integrate-and-fire, are documented in [19]. Table A1 in Appendix A discusses different criteria of interest regarding biological plausibility and their relation to neuromorphic hardware.

Canonical models are advantageous in studying the behavior of a single spiking neuron because they standardize the mechanisms of action potentials using the mathematical theory of dynamical systems. To investigate a network of neurons we must also model the neurochemical interaction and neural plasticity at the synaptic cleft of connected neurons, introducing certain challenges especially when concerning biological plausibility [17]. Together, populations of homogenous or heterogenous neurons may be connected to form a SNN and simulate its time evolution of neural activity. The resulting neural network fundamentally differs from an ANN where the input/output information and its process are not embedded in a temporal dimension. It is still an open problem how the information is encoded in a spike train, whether the frequency of spikes in a time window encodes information (rate-based) or the time between the spikes (spike-based). Brette suggests that rate-based encoding is a useful but limited ad-hoc heuristic for understanding neural activities and that spike-based encoding is more supported both empirically and theoretically [23]. For example, there are studies supporting interspike interval (a spike-based method) information encoding in the primary visual cortex [24].

**Figure 4:**
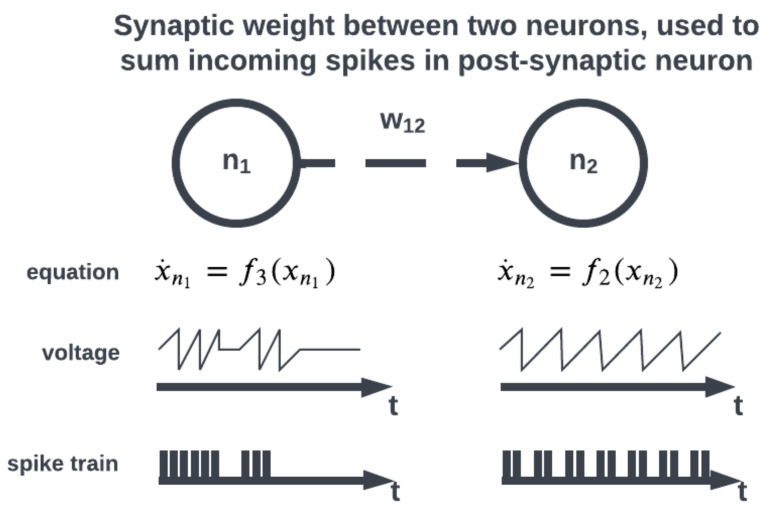
Incoming spikes between neurons are summed using a synaptic weight.

**Figure 5:**
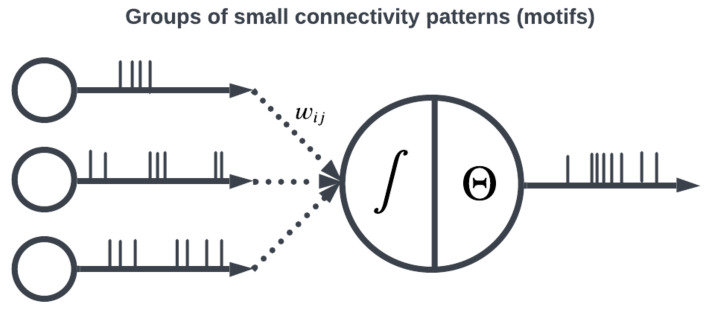
Patterns of connectivity are determined by weights between neurons.

**Figure 6:**
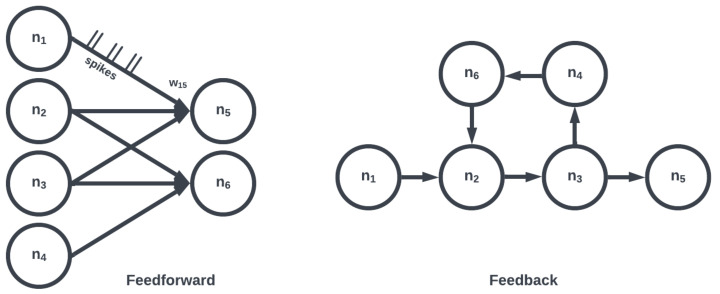
Large collections of motifs form layers or populations (e.g., convolutional, recurrent). Two networks consisting of the same number of neurons and edges may differ depending on organization.

The topology of a SNN prescribes how the neuronal activities are connected to each other by weights representing synaptic modulation between neurons (see Figure 4). The work of [25] showed that there are highly non-random small patterns of connectivity in cortical networks, an idea expanded upon more broadly for the brain by [26]. These non-random patterns between neurons are called motifs [27], an example of which is a typical convolutional (non-recurrent, feedforward) organization (Figure 5). Organizing sets of motifs, we may construct layers or populations of coordinated neural activities (Figure 6). The connectivity pattern and structure of the network influence information flow, signal processing, and the emergence of network dynamics. The choice of topology impacts how neurons communicate and interact with each other, affecting the propagation of spiking activity, synchronization, and formation of functional circuits. Different topologies, such as feedforward, recurrent, or hierarchical structures, offer unique computational capabilities. For example, feedforward networks excel at information propagation and feature extraction, while recurrent networks enable memory and feedback mechanisms. The topology of a SNN is a fundamental design parameter that shapes its computational properties and determines its ability to perform specific tasks, making it a key consideration in designing and understanding neuronal computation.

**Figure 7:**
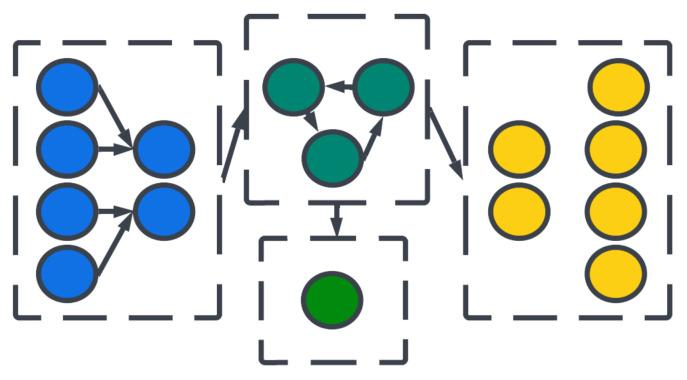
Organizations of neural layers and populations form architectures.

**Figure 8:**
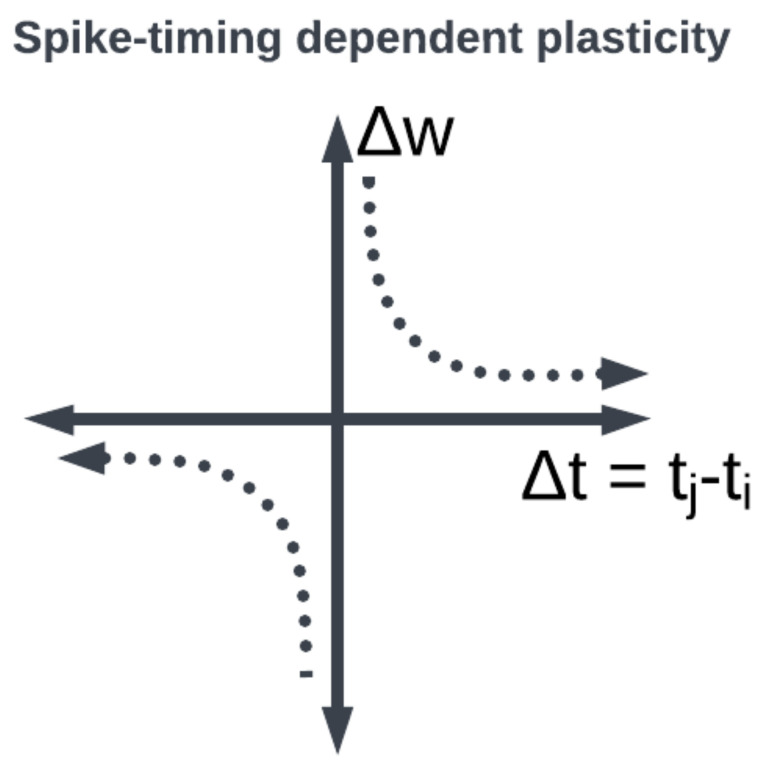
STDP synaptic learning rule window function: the weight between neurons is modified depending on when the pre- and post-synaptic neurons fired.

Large organizations of coordinated neural populations may be trained to perform AI tasks or approximate functions [28] (see Figure 7). In addition to the SNN topology, the adaptation rule that specifies the weights between interconnected neurons plays a crucial role in determining the computation performed by the network. Here, Hebb’s postulates, what are aphoristically understood as “cells that fire together wire together” [29], remains the dominant neuroscientific insight for learning. A seminal work in modeling synaptic plasticity, this is a well-known unsupervised learning technique called Spike-Timing-Dependent Plasticity (STDP) [30]. The STDP method works based on the selection of a learning window (see Figure 8) describing the asymmetric modification of the synaptic conductance between two neurons: when the presynaptic neuron fires before (after) the postsynaptic neuron, the conductance is strengthened (attenuated). The smaller the time interval between spikes, the greater the magnitude of modification in either direction. By applying a learning algorithm such as STDP to a population of spiking neurons, the learned (conductance) connection weights between neurons capture the topology of the network. A historical account of the developments from Hebb’s synaptic plasticity postulate to the STDP learning rule and its modern variations can be found in [31]. A survey of methods for learning via SNNs in [32] reveals that most learning models are STDP-based [33].

Alternative methods exist to learn synaptic weights, and their development is an active area of research for applications where biological fidelity may not be a priority. One such method is to convert ANNs to SNNs by mapping their weights [34,35,36]. Another approach is to use surrogate-gradient descent [37] such that traditional deep learning methods may be used by choosing an appropriate substitute for the spiking neurons’ Heaviside activation function. The substitute is typically a function that approximates the Heaviside function but has smooth gradients, such as the tangent hyperbolic function.

## 3. Neuromorphic Computing: Hardware vs. Software

**Figure 9:**
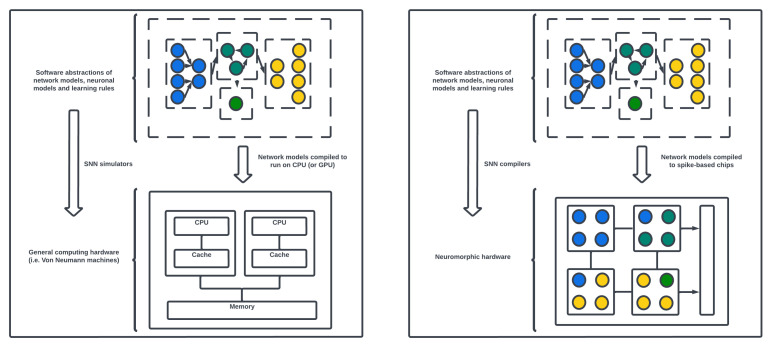
SNNs on general-purpose computing machines with a cache memory hierarchy that may be optimized to simulate the network efficiently, or SNNs compiled to neuromorphic chips to be run as in-memory computing.

SNN models may be simulated on von Neumann computing machines as well as compiled onto neuromorphic chips. Figure 9 describes the subtle difference between the two types of computing architectures and distinguishes where power gains can be made by utilizing the asynchronous event-based chips. Von Neumann computing, the traditional computing architecture, is based on the separation of processing and memory units, with sequential instruction execution and a focus on fast and precise arithmetic operations. In contrast, neuromorphic computing draws inspiration from the structure and function of biological neural networks. Neuromorphic systems typically feature specialized hardware that integrates processing and memory, enabling parallel and distributed computation with low power consumption. While von Neumann architectures excel at general-purpose computation and algorithmic tasks, neuromorphic computing emphasizes efficient and event-driven processing, enabling the simulation of large-scale neural networks and the implementation of brain-inspired algorithms. Neuromorphic computing offers advantages in areas such as real-time sensory processing, pattern recognition, and cognitive tasks that require high parallelism, low-power operation, and adaptive learning capabilities. Processors such as Central Processing Units (CPUs) and Graphics Processing Units (GPUs) are examples of von Neumann architectures. Often, GPUs (in contrast to CPUs) are used to accelerate the training and simulation of SNNs up to orders of magnitude by introducing parallel computing [38,39,40]. However, in practice, a heterogeneous system architecture consisting of some CPU/GPU and neuromorphic chips would be preferred [41,42].

Another option for efficiently implementing SNNs is simulating them on Field-Programmable Gate Arrays (FPGAs), often used when the application is low-power and not large-scale [43] or when real-time processing is a requirement [44]. FPGAs are reconfigurable integrated circuits that allow for custom hardware design and implementation through digital logic circuits, making them suitable for a variety of applications but requiring expertise to program them. FPGAs are particularly well-suited for implementing SNNs due to their ability to be customized at the circuit level. This feature allows for the deployment of small to medium-sized SNNs with reduced power consumption and enhanced performance through optimizing SNN computations, efficient parallel processing, and low-latency operations [45]. The work of [46,47] are two examples of SNNs on the order of thousands of neurons implemented onto FPGAs for real-time performance. Further development such as in [48] enabled the inclusion of an on-FPGA STDP for online learning [31]. Other works such as [44,46] investigated biologically plausible and biophysically meaningful SNN modeling on FPGAs. FPGAs may be seen as “in-between” typical von Neumann machines with more specialized hardware, similar to neuromorphic hardware, suitable for when a medium-sized CPU/GPU model needs accessing constrained resources.

Neuromorphic computing is an exemplary realization of In-Memory Computing (IMC) [49], particularly in the context of embodied cognition and robotics. Neuromorphic hardware, integrating memory and processing units, mimics the behavior of biological neural networks. Executing computations directly within memory reduces data movement, resulting in low-latency, energy efficiency, and parallel processing [50]. This approach offers substantial advantages for embodied cognition and robotics by enabling real-time, event-driven processing of sensory inputs, enhancing performance, and facilitating time-sensitive tasks. With efficient storage of synaptic weights and specialized memory technologies, neuromorphic architectures provide scalable and brain-inspired computing solutions. Tool development to evaluate this hardware is also an active field of research, one such example being the work of [51] which introduced SpikeSim, a platform for an end-to-end compute-in-memory benchmarking tool to compare different SNN models on a chip for their power and latency efficiency.

As neuromorphic computing platforms are still nascent and unavailable [52], the selection of software for simulating large-scale neural systems on digital hardware [53] is a decision contingent upon research needs, as a co-design problem [54]. Neuromorphic hardware is one type of specialized IMC hardware [55] capable of realizing certain classes and sizes of SNNs, while simulations run on general-purpose computers (e.g., CPU and GPU) require the numerical integration of sparsely coupled neuronal equations which are limited by memory and bandwidth constraints [56]. These limits can be overcome by a combination of software and hardware techniques, as in the work of [57] which makes use of code-generation for SNN [58], targeting GPUs; here, further software optimizations may be explored to accelerate SNN simulations on traditional hardware. Additionally, related work by [59] compiles models to be run on an emulated Loihi chip [1], allowing researchers to errorlessly simulate how their model would perform on the neuromorphic chip without actual access to one. This is possible because the Loihi chip can be shown to be equivalent to a leaky integrate-and-fire neuron with Euler time stepping integration [59]. Different neuromorphic chips implement different neuronal models by varying the design and functionality of their circuitry. Some chips may focus on implementing simplified spiking neuron models, while others may aim for more biologically realistic models, incorporating detailed features such as dendritic processing, learning rules, or higher-order dynamics, allowing for a wide range of neuronal behaviors and capabilities. The selection of neuromorphic hardware depends on the trade-offs in specific experimental or computational research, as different chips may offer varying compromises between computational power, energy efficiency, scalability, ease of programming, and support for different neuronal models. A non-comprehensive table listing neuromorphic chips and their tradeoffs can be found in Table A2, Appendix B.

Overall, the implementation of SNNs on different computing platforms presents distinct challenges and benefits, particularly in terms of the scale of neural networks that can be implemented [60]. CPUs provide flexibility in programming, making them suitable for small to moderate-sized SNNs. However, CPUs may struggle to handle large-scale SNN computations efficiently, resulting in slower performance and longer training times. GPUs excel in parallel processing and are capable of accelerating computations for medium to large-sized SNNs, although energy consumption may be a concern. With their high number of cores, GPUs can handle the massive parallelism inherent in SNNs, significantly speeding up training and inference processes. FPGAs offer low-latency and low-power operation, making them ideal for implementing small to medium-sized SNNs in resource-constrained environments [61]. FPGAs can be programmed to optimize SNN computations, resulting in efficient and high-performance implementations. However, FPGAs may have limited resources compared to CPUs and GPUs, which may limit the scale of SNNs that can be deployed [43]. Neuromorphic computing, leveraging specialized hardware like neuromorphic chips, provides an opportunity for implementing large-scale SNNs efficiently. Neuromorphic chips are designed to handle the unique characteristics of SNNs, allowing for the implementation of large-scale networks while offering energy efficiency and real-time processing capabilities. Therefore, the choice of platform ultimately depends on the scale of the neural network, available resources, and specific requirements of the application such as real-time computing.

Here, we compare two software platforms to highlight different types of interdisciplinary research needs: (i) Nengo [62], and (ii) Brian2 [63] (or Brian [64]), both of which are developed in Python [65]. For more information on different packages, consult with Table A3 and Table A4 in Appendix C that respectively compare various SNN simulators and SNN-based frameworks to support various studies, ranging from cognitive neuroscience to brain modeling. While simulators and theoretical frameworks are often developed in tandem (as in the case of Nengo), it is important to recall that these theoretical frameworks can be implemented in other libraries that may be better optimized for performance and efficiency. For example, a dynamic field theory (DFT) model was re-implemented in the Nengo library by [66]. Nengo is a functional-based simulator that includes a graphical user interface, allowing for interactive inspection and visualization of different values present in the neuronal population models. A user must implement their dynamical system equations in Python-like code within the namespace scope of the Nengo classes in order for the program to simulate the time evolution of the neural activities. These Nengo scripts may also be implemented without the interface as regular Python scripts when interactive model probing is not required. Brian instead uses code-generation [58] to transform a user-specified spiking neural network model to optimized code in Python (which also supports C++) for execution. The advantage of code generation is its high computational efficiency due to the generation of low-level codes for resource-intensive parts of the simulation cycle. While research is ongoing, both simulators are able to compile SNN models onto neuromorphic chips [67].

One constraint of Brian, absent in Nengo, is the implementation of dimensioned quantities. Brian applies the International System of Units (SI) for quantities declared in the model to check the correctness of the dimensionality of neuronal equations. This enforced sanity check of the physical consistency of neuronal models is advantageous especially when biological plausibility is a priority. However, whenever just the functionality of neural populations is concerned, the specific units of quantities may not be relevant. One example is the tuning-curve approach in Nengo, where the parameter values for a neural population are specified by a least-squares fit against the desired representational transformation for that population. Hence, as much as Brian is a suitable option for a neuroscientist who wishes to model biological neural networks, Nengo can help a roboticist reproduce traditional control systems with SNNs.

The high-level functional approach to brain modeling present in Nengo is particularly ideal in robotics applications, since the prescribed functionality (representation and logic) emergent in connectionist SNN models [68,69] allows for an abstraction of low-level neurochemical dynamics observed in vivo while achieving desired cognitive behaviors. This idea has been formalized using the Neural Engineering Framework (NEF) [70] and the Semantic Pointer Architecture (SPA) [71] that led to the development of the Spaun [72]. The NEF consists of three principles of neural computation: (i) a neural population represents a vector acting as a nonlinear encoding of spike trains and facilitating their linear decoding; (ii) various linear transformations of such vectors are defined by alternative decoding weights; and (iii) the neural representations can be treated as control-theoretic state variables. These three NEF principles allow for the vectorial representation of mental activities captured by neuronal and synaptic dynamics. The orchestration of encoding, transforming, and transferring vector signals among neural populations forms a connectionist model, where each population’s representation is a state variable in a control system and synaptic connections provide information feedback.

The SPA is an extension of the NEF that is based on the semantic pointer hypothesis [71] which states: high-level cognitive functions in biological systems are representable by operations on semantic pointers in a high-dimensional vector space. Semantic pointers are vector representations of neural networks that carry partial semantic contents and are composable into the representational structures necessary to support complex cognition. The mathematical method antecedent to the SPA, Holographic Reduced Representations (HRR) [73,74], allows for defining a convolutional algebra over a space of distributional (real-valued vectors) representations. In this algebra, two vectors representing partial semantic contents can be convolved to produce a third vector representing the binding of the partial contents. The addition of vectors is treated as a bundling of semantic representations. Both the SPA and HRR are examples of the Vector Symbolic Architectures (VSA), a survey of which can be found in [75]. Vector Symbolic Architectures are a class of computational models that use high-dimensional vectors to represent complex cognitive concepts, such as words, objects, or actions. These vectors can be manipulated using mathematical operations, allowing for the creation of novel concepts through the combination of existing ones. Table A5 in Appendix D describes the main features of VSA and their relation to SNNs. The work of [76] argued that VSA is an attractive approach to addressing problems in higher-order cognitive processes such as linguistics and analogical reasoning because they provide a way to represent and manipulate high-dimensional, distributed representations that are flexible and context-sensitive, while still preserving some of the key properties of symbolic representations. VSA has recently been generalized to the Vector Function Architecture (VFA) [77] which extends the algebraic operations on vector spaces to functional spaces. In this architecture, vectors not only represent individual data points but also elements of a functional space that can be bound, bundled, and unbound. The contribution of the SPA is to achieve a similar convolutional algebra but using vectors from the space of neural representations, determined by the SNN encoding tuning curves and decoding weights. In practice, this amounts to the projection of desired SPA vocabularies into a high-dimensional vector space such that the vector resulting from the convolution of two vectors is always orthogonal to those two vectors. This vectorial representation transforms a SNN model to a control system, where the vectors form the state variables; thus, the problem of neural process (cognition) becomes a control problem of how to transition neural activities from one state (representing some semantic content) to another. This transition can be a complex combination of convolutional bindings and vector additions but the resulting vector nonetheless stays in the state space of the system. For example, a list structure in working memory can be realized by binding semantic contents to different semantic pointers and bundling those vectors together as a neural representation of the memory. Then querying a specific content is the deconvolution with the desired pointer that zeros all other semantic vectors in the binding due to the orthogonality property. As seen in the approach taken by NEF, constructing spiking neural networks whose spiking activities code the vector symbolic operations allows for the construction of higher-level cognitive algorithms capable of being deployed on energy-efficient neuromorphic hardware. SNNs are thought to be efficient for their exploitation of spatiotemporal sparsity: i.e., at any point, not all neurons are active, and therefore the modeling and hardware implementation of these networks may similarly be massively parallel and asynchronous. Thus, the realization of a VSA using SNNs suggests that spatiotemporally distributed spikes are able to encode the operations performed by conventional VSAs in a power-efficient manner. Table A6 in Appendix E summarizes different parallelisms possible in SNNs.

## 4. SNN Embodiment into Cognitive Robotic Systems

A robot is conceptualized as an information-processing machine capable of planning (guidance), perception (navigation), and control of its physical actuators. Such machines would be imbued with physical bodies embedded in a sensed environment, with some representation of their body in the form of kinematic and dynamic models (differential equations) that are used in the robot’s GNC system and accounted for in assigning goals and future plans. The human–robot collaboration in complex tasks was analyzed by [78] as an ethical design problem, where trust must be built between society and robotics by designing robots that adhere to social norms. Before reaching this turning point, much progress must be made to improve the “true” adaptability and autonomy of robotic systems. Three crucial steps toward this goal are: (i) biological capitulation of the traditional robotic perception-planning-action cycle, (ii) embodiment of self-aware (i.e., proprioceptive and exteroceptive) agents in an environment, and (iii) reconfigurability of the embodied self-aware agents. In the following, we review the application of neuromorphics in robotic sensory data processing and, as an example with the most body of work, we focus on dynamic cameras. We then close the cognition loop over the robotic perception-planning-action cycle by carefully considering the interrelation between neuromorphic processes and their cognitive interpretation at the intersection of computational neuroscience and cognitive science. We claim that the establishment of SNN-based brain EEG analyses can further contribute to understanding this interrelation and explainability of SNNs, by connecting the SNN activities to human behaviors. Hence, we dedicate a section to critically overviewing the existing methods for SNN-based brain analysis.

### 4.1. SNN-Based Vision for Robotic Perception

Sensory data processing plays a critical role in robots’ perception of their body and the environment they are embedded in. Vision is an important perceptual feature of many cognitive tasks, and SNNs provide a way to biologically [79] present a retina-like SNN for image reconstruction. The authors of [80] make use of known areas of the brain along the human vision pathway to construct a topologically analogous SNN that models spatial and visual mental imagery. Similarly, [81] organizes its neural architecture based on known anatomical structure, but specifically details the lateral geniculate nucleus [82], a subregion of the thalamus involved in vision, treating other neural populations as retinal neurons or interneurons. Such large-scale network models are useful for ablation experimentation where lesions (e.g., reducing the number of neurons in a population, inhibiting synaptic dynamics, etc.) at points along the pathway are known to produce patterned artifacts in the reconstructed image, and therefore may be compared to empirical evidence. This can be seen as a perturbation of model parameters to observe changes in the model behavior (e.g., how is the perceived visual field affected by reducing the number of neurons along the visual pathway? Or, how is the performance of a task affected by changing the neuronal model?) [83]. Mesoscopic-level perturbations include lesioning areas represented by anatomical subnetworks/modules, such as the retina, to observe the degraded performance of image reconstruction. As many large-scale simulations make use of simplified neuronal models in order to be computationally tractable, research on the lower-level (contrary to mesoscopic) perturbation of SNN models includes making alterations to the neurons’ model.

**Figure 10:**
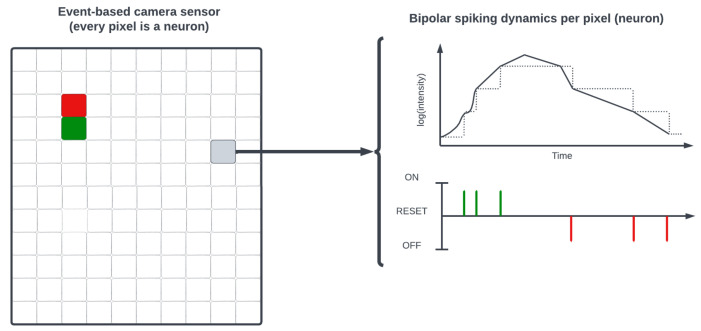
Event-based camera sensors treat each pixel as a neuron sensitive to the log change in photon intensity. This form of sensor is comportable with SNN models for image processing because a spike train may be read out from the sensor as input to downstream populations.

Artificial photosensitive spiking retinal networks are the fundamental concept used in a new generation of sensors called event-based cameras or dynamic vision sensors [84]. Table A7 in Appendix F provides a non-comprehensive list of currently available dynamic vision sensors. Event-based cameras encode visual information as discrete events (e.g., changes in light intensity) concurrently over a field of independent photosensitive neuronal models and require spiking algorithms to reconstruct and process the image (see Figure 10). For this reason, SNNs are comportable with these event-based retinomorphic sensors that more closely capture the photosensitive action potential dynamics of biological vision systems. A survey of the developed specialized spike-aware algorithms for image processing and depth estimation by event-based cameras can be found in a recent review paper [85].

**Figure 11:**
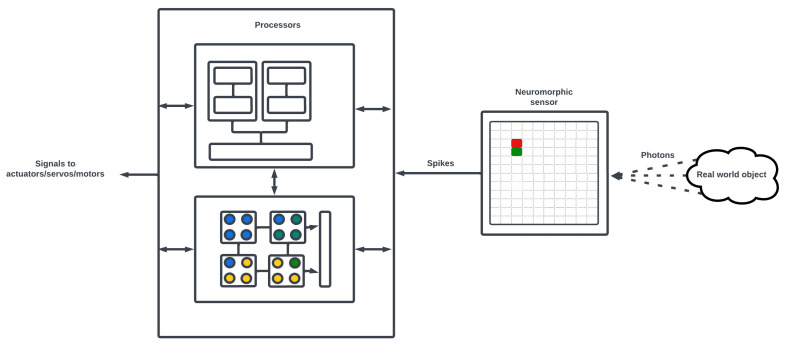
The deployment of neural models for event-based sensing may offer a power-efficient way to process large streams of data in real time by only processing data as changes (to both proprioceptive and exteroceptive senses) arrive.

The exploitation of event-based image representation is demonstrated in [86], where two streams of event camera data are passed into a SNN architecture composed of two cooperative populations (one for coincidence and one for disparity) to produce instantaneous stereo depth perception with real-world stimuli. The work of [87] similarly uses two spiking neural populations connected to two neuromorphic cameras to solve the stereo correspondence problem. Other traditional computer vision tasks, such as optical flow, have also been adapted to event-based sensors to perform flow estimation in a spike-driven manner. It has been demonstrated that for event-based optical flow estimation, an SNN-based self-supervised learning approach outperforms traditional supervised methods and achieves state-of-the-art performance [88]. Developing/training algorithms for image processing requires access to data from event-based cameras, which can be prohibitively expensive; thus alternatively, we can use preexisting datasets with estimated ground truths. The dataset of [89] presents an event-based dataset for pose estimation, visual odometry, and other related tasks. Another example of such datasets is the Multi-Vehicle Stereo Event Camera (MVSEC) [90], which provides event-based data for 3D perception tasks. The authors of [91] take advantage of this dataset to develop a framework, called StereoSpike, that performs depth estimation using SNN-type neural processing in a UNet-like encoder-decoder architecture. For spiking data or networks, rather than a single pass of information as in a convolution, the network is always “on” and processing changes to the current neural representation as opposed to recording raw values in conventional camera systems. This event-based processing of data provides gains in energy efficiency as the processor is effectively doing no task while there are no changes to the scene (see Figure 11).

Beyond event-based datasets, conventional image and video datasets are converted into spike trains using spike encoding and can be benchmarked with SNNs. Datasets such as the Middlebury Stereo Dataset [92] have been encoded into spike trains to be used as SNN inputs for static image tasks. For this purpose, different spike-encoding schemes can be applied, namely, rate coding, temporal coding, and rank-order coding, that offer distinct approaches to represent information in spiking neural networks [93]. Rate coding relies on the firing rate of neurons to encode information, where higher rates indicate higher stimulus intensity. This scheme is simple to implement and robust to noise, but it lacks precise timing information. Temporal coding, on the other hand, encodes information in the precise timing of spikes, leveraging the relative timing of spikes across multiple neurons to convey information. This scheme enables precise temporal resolution and can capture fine-grained temporal dynamics but it is more sensitive to noise. Rank-order coding represents information based on the relative order of spike timings among a group of neurons. It provides a distributed representation that is resistant to noise and changes in firing rates, enabling efficient coding and decoding of information. However, rank-order coding requires more complex decoding algorithms. The choice of spike-encoding scheme depends on the specific requirements of the task at hand, considering factors such as temporal precision, robustness to noise, and the nature of the information being encoded. Such schemes are important for bridging and testing traditional image processing approaches in a spike domain according to the application.

Given the spike encoding of two signals (or even two different encodings of the same signal), it is instrumental to have metrics for comparing the two sets of spike trains. These metrics capture various aspects of spike timing, such as spike rates, spike timing precision, temporal correlations, and spike train regularity [94,95]. Considering these metrics, researchers gain insights into the dynamics, synchronization, and information processing of neuronal populations, facilitating the understanding of neural coding, network connectivity, and computational mechanisms underlying neural systems. For this reason, applying spike encoding to existing real-world datasets and benchmarking them using various spiking metrics can potentially aid interdisciplinary collaboration [96]. This signifies the cooperation of various fields, e.g., neuroscience, computer science, and engineering, to provide common frameworks for evaluating SNN models, develop standardized benchmarks, promote knowledge sharing, and foster advancements in neuromorphic computing toward more accurate and efficient models aligning with realistic neural dynamics. Table A8 in Appendix G reviews some metrics that are utilized to compare spike trains in different applications.

Vision is a complex process involving the integration of various neural processes that allow us to perceive the world around us. It is an excellent example of how the brain processes information and makes sense of our surroundings. The biomimicry, evident in an event-based sensor, is another example of how a co-design between biological fidelity and artificial perception can advance image processing and our understanding of the sense of vision. Many other senses, i.e., hearing [97], touch [98], smell [99], and taste [100], are endowed in the human being, whose received information is fused (or integrated) in our brain to have a better perception of our environment. Two examples of such correlated senses that have been subject to research in the field of computational neuroscience are smell [101] and touch [102]. The work of [102] models the cochlea, an organ used for hearing, using a model of the sense of touch to simulate the behavior of the mechanical waves that propagate along the basilar membrane of the cochlea. The sense of touch model is based on the idea that the basilar membrane can be captured as a bank of bandpass filters, each tuned to a specific frequency. As a result, the model reproduces the frequency-selective properties of the cochlea and the auditory nerves. Similarly for a robot, having the ability to integrate correlated information from different sensors is crucial, especially in environments where one sense may not be sufficient. By mimicking biological systems, robots could potentially use multisensory integration to navigate and interact with their environment more effectively. Therefore, further research on the neural processing of other senses can lead to the development of more advanced and sophisticated navigation systems for robots.

### 4.2. Neuromorphic Computing for Cognitive Robotic GNC

A crucial aspect of every robotic GNC system is the Simultaneous Localization And Mapping (SLAM) [103]. SLAM is a technique used in robotics, often with the help of computer vision, to construct a map of an unknown environment and at the same time locate the robot within the environment. It addresses some of the challenges of autonomous navigation of robots without relying on pre-existing maps or external positioning systems. SLAM involves algorithms that utilize various sensors to gather data about the environment over time and fuse their provided information to estimate the robot’s trajectory and create a consistent map. SLAM has a broad spectrum of applications in autonomous robots, self-driving cars, augmented reality, and virtual reality systems, enabling them to explore unknown environments, build accurate maps, and localize themselves in real time [104]. At a philosophical level, SLAM aligns with embodied cognition by incorporating the physical embodiment and environmental interactions of a robot. By considering the robot’s embodied experiences and interactions, SLAM provides a concrete implementation of embodied cognition principles in the domain of robotic perception and spatial awareness. The use of SNNs for SLAM problems on neuromorphic chips is an active area of research [105,106], especially as perceptual sensors and models of sensory integration improve. SNNs can process sensor data to extract features and recognize objects, while their temporal and parallel processing capabilities enable efficient representation of sensory information. SNNs can model temporal dynamics and incorporate memory mechanisms, aiding in the temporal integration required by SLAM algorithms. Furthermore, SNNs excel at handling event-based sensor inputs, allowing SLAM systems to process asynchronous and event-driven data more effectively. The work of [107] proposes an architecture where SNNs process sensory data, such as camera images or LiDAR scans, to estimate the robot’s pose and simultaneously create a map of the environment. They demonstrate the effectiveness of SNNs in capturing temporal dynamics, handling event-based inputs, and incorporating adaptive learning mechanisms for improved SLAM performance. By incorporating adaptive navigation and learning mechanisms, SNNs enable robots to improve SLAM performance over time, while the low-power operation of neuromorphic chips designed for SNNs promotes energy-efficient real-time processing. The integration of SNNs into SLAM represents a promising approach for developing more intelligent and autonomous brain-inspired GNC systems for robots. One important example of using biologically inspired organization for SLAM problems is that of the rat hippocampal model called RatSLAM [108]. Later work re-implemented the model using the NEF and Nengo in order to realize the algorithm on spiking architectures [109].

GNC plays a critical role in enabling autonomous systems to perceive their environment, make decisions, and execute control actions. A navigation system (e.g., SLAM) provides the necessary spatial awareness, while the guidance and control modules ensure accurate planning and task execution to achieve desired goals. It is at the intersection of navigation and control that SNNs may be useful for “closing the loop” between perception and action. As demonstrated by models such as NeuCube [110], SNNs are able to integrate large amounts of spatiotemporal data from different types of sensors; and as demonstrated by NEF, SNNs are also able to form control systems to be connected to sensors and actuators. One attempt to close this loop that claims to be the largest functional model of the brain (attached to a robotic arm) is called Spaun [71]. Spaun is a SNN-based platform, which replaces the conventional robotic perception-planning-action model with a perception-cognition-action model of biological cognition to perform cognitive tasks such as the Raven’s Progressive Matrixes assessment using a simulated robotic arm. One of the notable features of Spaun is its ability to perform multiple cognitive tasks, including pattern recognition, working memory tasks, and problem-solving. This attempt is beyond simple learning of pre-specified control functionalities by a SNN to produce the same input/output of a traditional controller. Instead, Spaun encodes every signal in the system, including memory, sensory input (e.g., from the vision system), and control output (e.g., the voltage at actuators), into spike trains. At its cognition core, then it uses semantic pointers to convolve the spiking neural representations of signals and make decisions. This contribution of introducing cognitive functionality in SNNs however, does not account for low-level neuronal biological plausibility, since the Spaun employs leaky-integrate-and-fire type neuronal models. Further work extending Spaun with biologically plausible neuronal models was performed in [111] where compartmental neuronal models were used to capture the effect of the neurotoxin tetrodotoxin. Degraded performance was observed wherein the agent “forgot” what task they were doing while perceptually still processing input stimuli.

Neurally coded VSA-based cognitive models such as Spaun could potentially be used in conjunction with SLAM and other modules of GNC systems in advanced autonomous systems. For example, cognitive models can inform decision-making processes or provide higher-level cognitive functions for robots navigating and interacting with complex environments. This integration would go beyond the core concepts of GNC and involve additional layers of cognitive modeling and processing that can be implemented by SNNs running on neuromorphic chips or FPGAs in order to perform in real-time.

Michaelis et al. presented an algorithm for training a SNN to do robust trajectory planning on a Loihi neuromorphic chip [112]. The work made use of neuronal motifs to initialize and learn the features required to generate complex robotic movements. However, in comparison to Spaun, such networks lack the ability to reason with higher-order cognitive structures as in VSAs. The compromise between high-level cognitive functionality and low-level biological fidelity characterizes a spectrum of design choices when modeling cognitive systems using SNNs. At either radical end, there are designs of self-aware agents that constantly construct a representative model of the world to compare against the sensed environment to effectively adapt to new situations, in coordination with their goals [113,114]. The difference between NEF and SPA also demonstrates this spectrum, where NEF may be useful as a signal processing approach for sensory integration and SPA makes use of this neural signal processing to support vector representations of semantic content for higher-order cognitive tasks. These semantic pointers can be used (as in any VSA) to represent complex hierarchical data structures for navigational robotic tasks. Neurobiologically inspired self-monitoring systems are described in [115] and their principles of information processing at different levels, including low-level sensorimotor control, maintaining homeostasis system set points, and high-level cognitive planning, are studied. Self-awareness here refers to cognitive systems with proprioceptive and exteroceptive senses.

### 4.3. Brain Analysis and Explainability of SNNs for Cognitive Robotics

Our perception and awareness are facilitated through sensory integration. Once external stimuli are encoded into neural activity by perceptual organs, the coded information is continuously combined and fused by parts of the brain. It is, therefore, important to have a fine understanding of the dynamics of the cortical and subcortical regions of the brain. EEG is a method of recording electrical activities at the brain’s surface over time (up to one-millisecond temporal resolution) that is measured by nodes distributed across the scalp. The biological and mathematical interpretation of what EEG signals represent and the processes that produced the phenomenon is not a settled matter in neuroscience [116,117,118]. The essential crux is formulated as an instance of the neural inverse problem [119]: Given a set of observed continuous time-varying vector signals representing multichannel EEG, what neural process models, inputs, and parameters reproduce this observation? The solution to the neural inverse problem specific to EEG may be evaluated along the theme of biological plausibility, i.e., how accurately the model captures the biological elements of the observed brain activities. This type of brain analysis can significantly contribute to our understanding of the relation between SSN activities and the emerging behavior of a robotic agent equipped with neuromorphic computation, and consequently to the explainability of SNN activities. In the case of abrupt changes in the human body due to, e.g., injuries or disabilities, this analysis also helps develop effective neuroprosthetics and decode the brain plasticity to adapt fast to a new condition, which can be instrumental to the brain-inspired GNC of reconfigurable robotic systems.

In EEG signal classification tasks, where certain features of the signals are extracted and labeled, the question of biological plausibility is not necessarily of immediate importance as a neural network need only classify an EEG recording [120]. However, it is argued that biologically enhanced models improve monitoring performance. As an example in monitoring epilepsy, EEG signal classification may be a statistical calculator to predict whether a patient will have an episode or not, and a classifier designed predicated on a model of brain elements promises to faster converge at more accurate solutions. Traditional EEG signal processing techniques vary but remain in the realm of transformations for feature extraction in spectral space. For this purpose, beyond classic Fourier methods, wavelet [121,122,123] and fractal-based methods [124,125,126] have been used. The biological plausibility of EEG signal processing is often claimed by using SNNs in the process. The SNN-based techniques of EEG signal processing rely on spike-encoding algorithms to transform EEG signals into a spike train such that the information is readable by SNNs. See [127] for a systematic analysis and guideline for SNN-specific encoding methods and error metric selection.

The Bens Spiker Algorithm (BSA) [128] is one example of a lossy encoding where a finite impulse kernel is deconvolved with the signal to extract time-specific frequency information as spikes. BSA has been used to classify EEG by means of an evolving probabilistic SNN whose neurons were merged according to the Euclidean distance of their parameters [129,130,131]. Although similar to traditional approaches in lossy encoding we must still select some parameters such as the kernel and the bandwidths of neural oscillations of interest, this comportable technique with SNNs enjoys a model of the in vivo processes generating the EEG signals. Accordingly, the spatiotemporal properties of brain data can be injected into the modeling and feature extraction. Kasabov et al. introduced the NeuCube [110] SNN architecture for brain-like AI by combining these spike-encoding and evolving SNN topology modules in an EEG classification task. The NeuCube belongs to a class of SNN models called liquid state machines taking advantage of reservoirs of highly recurrent motifs. The initial topology of the SNN reservoir module, i.e., memory repertoire, is obtained by solving the EEG localization problem and it is dynamically evolved based on an STDP-derived learning rule. This framework has been extended to include Functional Magnetic Resonance Imaging (fMRI) data for a similar classification task [132] as well as personalized predictive modeling making use of this spatiotemporal fMRI-EEG data integration [133]. An application of NeuCube can be found in [134] which applies these techniques and uses learned network weights as a discriminating feature to predict the effectiveness of mindfulness therapy on depression. A different type of EEG data integration using NeuCube was conducted by [135] which included audio-visual stimulus data for short-term emotion recognition. Neucube is a valuable tool in neuroscience research due to its ability to integrate different types of spatiotemporal brain data. By combining data from various sources such as EEG (measuring cortical electromagnetic activity) and fMRI (measuring metabolic consumption and functional network regulation), Neucube provides a comprehensive multimodal framework to study the brain’s spatiotemporal dynamics. This integration enables researchers to gain a more holistic understanding of brain activities, uncover complex patterns, and explore the relationships between different brain regions and their dynamics. The NeuCube’s capacity to assimilate diverse brain data facilitates interdisciplinary collaborations and advances our understanding of brain function, cognition, and neurological disorders.

The NeuCube algorithm is an example of a SNN trained to do classification and implemented on traditional computing machines, i.e., there is no software currently available to compile NeuCube to an existing neuromorphic chip. Developing software tools to compile existing algorithms onto neuromorphic chips is an open problem at the intersection of biological network simulation and SNNs for AI. The NEural Simulation Tool (NEST) is an example of a well-established attempt at software standardization deploying a markup language to represent high-level specifications of neuronal dynamics and connectivity that may be run by a neural simulation engine (e.g., Brian or Nengo). Though given the diversity of the available hardware and software, interoperability is a challenge at multiple levels: (i) different neuromorphic chips require different compilers, (ii) models with various learning rules and organizations must be supported across neuromorphic chips, (iii) different degrees of software abstraction is implemented in the neural engine pipelines. These challenges must be addressed before a robust economy of neural description, simulation, and compilation tools can take hold to facilitate interdisciplinary research in brain sciences. The work of [136] is an early example of addressing these problems using analog spiking hardware with software agents for real-time spiking systems. Later work such as [137] further demonstrates that hardware/software co-design of neuromorphic systems is key for realizing large-scale simulations.

The NeuCube and NEF are considered two separate computing reservoirs with design decisions constrained by research goals. While NeuCube dynamically evolves its reservoir neuron weights using STDP-like learning rules, NEF determines neuron weights by decomposing the weight matrix into a non-linear spike encoding and a linear decoding that convolves these spikes with the neuron’s post-synaptic current in order to be integrated by the soma. The advantage of NEF is that firing rates and stimuli sensitivity may be prescribed for a neural population and an encoder/decoder weight optimization problem is solved in order to produce neurons that behave in a prescribed manner [70]. This allows the NEF to implement behavioral simulations where different tests may be run with the number of neurons in a population changed to observe the difference in encoding/decoding accuracy of an input stimulus. These tests would represent the same dynamical system of neural representational state variables being computed with varying levels of neural resources. Ultimately, both frameworks target different use cases: NeuCube can be thought of as a SNN-based machine learning algorithm for spatiotemporal data of any kind including brain data, whereas NEF is a way to design cognitive systems using neural population coding and design heuristics such that it may be falsified against real-world recorded data. Where an in vivo experiment collects spiking data, the NEF may be used to construct a network to perform the same task as in the experiment in order to compare the spiking patterns observed in nature and the spiking patterns generated in the simulation [71].

Studying brain activities not only can unravel mysteries about its efficient processing capabilities and cognitive behavior but also can provide solutions, even in clinical practices, for the challenges of disembodiment, e.g., in Spinal Cord Injuries (SCIs) [138]. There is strong evidence supporting that SCI immediately changes the state of the brain by producing extensive long-term reorganization of the cerebral cortex [139]. Recent results show that low-frequency EEG in persons with this type of injury can decode attempted arm and hand movements [140] leading to the development of intuitive EEG-controlled grasp neuroprostheses [141]. An implantable Brain-Computer Interface (BCI) for neuroprosthetics has enabled volitional hand grasp restoration in a human subject with complete cervical quadriplegia [142]. Although wearable neuroprosthetics are known to extend bodily functionality to assist people with SCIs, only a small number of patients use these devices mostly due to a lack of their appropriate embedded representation in mind. A new study highlights the homeostatic role of autonomic and interoceptive signals and their possible integration in a personalized experience of exoskeletons with the aim to facilitate personalized user-centered robotic technologies, which could overcome the hesitancy toward wearable neuroprosthetics [143]. By leveraging SNNs, researchers and clinicians have explored innovative approaches to facilitate communication with the neural system and control of neuroprosthetic devices, for individuals with limited motor functionalities [144]. The neural data recorded from a 96-electrode array in the premotor/motor cortex has been decoded using a SNN, in the task of point-to-point arm movement [145]. In another work, the authors propose a SNN-based approach using surrogate-gradient descent learning to reconstruct and generate multi-class artificial EEG signals from just a few original samples, which was used for BCI [146]. A methodology has been developed in [147] that is based on the NeuCube framework to extract deep knowledge and structural patterns from spatiotemporal data, using SNNs. It is claimed that the computational framework is well-poised to unveil the topological patterns of the brain and enhance the state-of-the-art in brain-inspired BCI. A novel brain-inspired SNN model for incremental learning of spike sequences has also been proposed that maps spiking activity from input channels into a high-dimensional source-space [148]. This model is applied to successfully predict continuous muscle activity and kinematics from EEG signals during upper limb functional movements.

### 4.4. Towards Self-Aware Reconfigurable Cognitive Robotics

The notion of neurorobotic self-aware agents invokes the notion of embodied cognition: one cannot have a physical agent without accounting for its environment to interact. Indeed, intelligence may be defined as the adaptable goal-oriented interactivity of the agent with the environment. Returning to the discussion of computationalism and the substratum of computation, robotics offers neuroscience research a platform to study embodied cognition in the context of self-aware systems. Ziemke discusses the role of robot simulations in embodied cognitive science, emphasizing the brain/body/environment conceptual triad and arguing that this systematic approach is particularly useful for active adaptation in non-trivial environments [149]. Embodied cognition has found support within the neural information processing community. Both the Neural Field Theory (NFT), a specialization of the dynamic field theory to neural tissue activities [150], and NeuroConstructivism (NC) [151] investigate aspects of embodiment. The NFT is a theoretical framework for representation-in-the-moment that is grounded on the theory of dynamical systems and neurophysiology, whose features can also be traced in Nengo [66]. The embodiment of the NFT is derived from the use of neural computation for various (embodied) cognitive tasks, e.g., stabilizing working memory, coupling sensory/motor dynamics, intentionality, and autonomous learning. Further examples of the deployment of the NFT in the construction of embodied cognitive systems may be found in [152,153,154]. The NC is a theoretical framework accounting for the evolutionary development and ontogeny of neural systems [155] consisting of several levels of organization, including encellment of neurons, enbrainment of neuronal networks in the body, and embodiment of the body in the environment. Both frameworks include accounts of neurophysiology in various scales. The NFT takes a field approximation at the scale of tissue and the NC takes a more comprehensive gene-to-cells-to-body-to-environment approach, thus they differ in their specific treatments of SNNs as dynamical systems.

Neuromorphic computing, with its ability to efficiently process sensory information and perform real-time computations, can overcome computational constraints by enabling the integration of sensorimotor feedback loops and facilitating the embodiment of cognitive processes in robotic systems. The relation between neuromorphic computing and embodied cognition lies in their shared focus on the integration of perception, action, and cognition. Neuromorphic computing can provide a platform for implementing and studying embodied cognition models. By emulating the principles of neural processing and real-time interaction with the environment, neuromorphic systems can support the embodiment aspect of cognition, allowing agents to sense and act upon their surroundings in a manner more akin to biological organisms. Assuming a biologically plausible model of the perception-cognition-action cycle realized within a self-aware robotic body, a robot adapts to its environment in different fashions. It may change its high-level cognitive strategy for achieving its goal, or if possible (depending on the implemented body) enact a transformation of its body to adjust in tandem with this strategy. Robots with such transformation capabilities are called reconfigurable robotic systems [156]. Self-reconfigurability is a desired property of an embodied agent as adapting one’s own physical constraints to a situation at hand is a feature of intelligence, particularly when that agent is self-aware of and sensitive to its embeddedness in the environment. Traditional robotic control systems have been recently implemented for self-reconfigurable robots. For example, [157] uses a robust adaptive fuzzy controller to adapt a dynamic self-reconfigurable robot to different configurations without adjusting control parameters. Table A9 in Appendix H compares the main computational approaches for four categories of robots. There is a progression of adaptability in the history of the development of robotic systems, where traditional robotics can be seen as the least adaptive because the implemented control systems were typically not able to handle changing environments. Reconfigurable robots are able to respond to these changes in their environment and they may use SNNs to do so. However, most adaptive approaches using SNNs are not well understood in the same way control theory is applied to traditional robotics. Frameworks, such as the NEF, help advance toward a more rigorous mathematical treatment of spiking dynamics within a feedback control system consisting of different modules of a GNC technology.

## 5. Coda: Trends, Limitations, and Prospective

In this paper, we have examined that striking the right balance between computational efficiency and biological realism is one of the challenges at the intersection of AI, robotics, and neuroscience, and there are ongoing debates on the accurate representation of neural processes versus the efficiency of network dynamics. At present, many of the debated points focus on the characteristics, limitations, and synergy of the hardware and the software. It can be said that initially biological plausibility was frequently sacrificed for computational efficiency, due to the fact that neural networks are basically a simulation of analog parallel processing on traditional Turing machines. In this sense, our endeavor to use neural networks has been nothing but an in-principle demonstration of what could be possibly performed given the limitations of the underlying hardware, which is not truly an analog to nervous tissues.

Therefore, in this first historical phase, the contribution of the research on neural networks and especially experimenting with deep networks have been mostly on the algorithmic side. To further the applications on complex systems embedded in challenging environments, the dominant AI research community awaits new advancements in quantum computation and hypercomplex multidimensional dynamic systems, whose formal transparency is the main dispute, which reaffirms that we are still dealing with implications of Godel’s incompleteness theorem. This de facto has been sidestepped in the last ten years as neural networks have been progressively exploited independently of their designs, given increased computational efficiency, speed, and power in this era. We have witnessed an explosion of hidden layers from single digits to over 50 in deep learning research and applications. However, there are no 50 layers of neurons anywhere in the brain, so any tenuous biological link is lost in such most recent developments. This state of affairs has an enormous downside in that these new networks create increasingly complex emergent computational dynamics, while scientific explainability and transparency tend to decrease asymptotically. It might be refreshing to use an example that is already old news in the field of AI: the complete domination of AlphaGo software [158,159,160] in board games, already exhibiting behaviors that are perceived by champion gamers as “incomprehensible” and “alien” [161].

In the last five years or so, it appears we have entered another historical phase of research on brain-inspired intelligence with the birth of neuromorphic hardware. Such hardware is designed starting from the principles of the material emulative constraints of neural tissues, such as memristive devices [162]. As we have discussed in the previous sections, in this instance, the driving force of computational efficiency is increasing adherence and fidelity to biological reality (although as we argued ”reality” is still an abstract model). Even so, as our review suggests, the challenge of designing fitting software remains the same as in non-neuromorphic devices. As we have mentioned, the current practice is to develop algorithms that are adaptable to neuromorphic hardware and then test them. The issue of explainability is not resolved yet but simply redistributed.

We have discussed how some of the issues involved in biological plausibility have been dealt with in some of the applications, considering robotic vision as a paradigmatic example. This particular choice was dictated by the fact that there is a wide consensus that vision is one of the most and best studied and understood processing modalities in current computational neuroscience research [85]. There are now several reviews that have addressed theory and application from the point of view of artificial intelligence and one of the trends has been to approach issues of explainability and transparency by reference to what we know about the brain and neural processes in vision [80]. This, however, presents some of the same limitations we have raised regarding assumptions of biological plausibility given incomplete and debated knowledge in neuroscience. That is, we still do not know many details about vision as a biological process and less in relation to incorporating it into behavioral functions. Neurological facts are still maps (models) of reality rather than the thing itself (the territory).

An additional limitation in the current literature is that the applications are very specific and modular and rarely link, e.g., two different sensory modalities or cognitive functions. While it is important to consider the generalization of findings and methodologies to other cognitive functions and domains other than vision, this is performed in principle and very few applications and implementations transcend the original modules. We have highlighted some of the tools already available in the literature that could potentially be used to lay these bridges across modalities and functions.

In terms of the state-of-the-art, the marriage between biological plausibility and computational efficiency has no real compelling grounding. We argue that this changes when the relationship is referenced to embodied agents such as robots. The reason for this is that explainability and transparency can be shifted from the purely computational and algorithmic level to the behavioral and cognitive level. As Chen et al. [163] argue, neurorobotics follows similar methodological logic to the field of neuroethology. Neuroscientists are faced with similar problems in understanding why some systems behave the way they do. The neuroethological approach attempts to address this issue by closely observing behavior while recording neurons or manipulating brain circuits. In a similar way, neurorobotics can be used to explain how neural network activities lead to a certain behavior. We submit that cognitive neurorobotics provides so far the best grounding to integrate biological plausibility, computational efficiency, and behavioral explainability. It further provides the best testbed to examine the ethical issues in interpreting emerging complex deep neural networks and machine learning behaviors. As neuromorphic systems become more complex, the interpretability of their behavior might become a challenge. Understanding how these systems make decisions and behave in complex real-world scenarios will be non-trivial.

## 6. Conclusions

A key research question lying at the intersection of neuroscience and AI concerns the degree of biological plausibility of the models used to capture cognition in artificial or living systems. Inevitably, due to their biomimicry roots, SNNs play a crucial role in any research aiming to either explain the neuroscientific behavior of biological specimens or provide insight into modeling their cognitive behavior. In this paper, we provided a brief tour of relevant topics to highlight the significance of SNNs in a wide variety of fields from the analysis of brain activities to embodied cognitive behavior of robotic systems. We visited the everlasting tension between the biological plausibility and computational efficiency of neural models and argued that this conflict may have been seized by the invention of neuromorphic hardware. We documented that the existing literature supports the use of SNNs for even traditional EEG signal processing (classification) tasks, which can still benefit from neural modeling of the processes generating the data, notably, to simplify the neural inverse problems. Utilizing these models for neuroprosthetic devices and their integration with brain-computer interfaces were also highlighted to bold the transformative impact that these technologies can have on restoring functionality and improving the lives of individuals with limited motor functions. Where there is a need for capturing the neuronal behavior of biological objects, e.g., in the classification of canonical neuronal models, the increased efficiency of information processing may indeed be taken as further evidence of the computational validity of biological models or as neurocognitive mimetic improvement for an artificial agent. Further, we argued that robotic agents are suitable platforms for testing hypotheses in the field of embodied cognition, due to the availability of the sensing, commanding, and processing information in a physical engineering system. Such a testbed should be also ideal for improving the intelligence, including adaptability and self-awareness, of the robots’ GNC systems. Therefore, it is here that SNNs offer a theoretically robust (leveraging the NFT and the SPA) and computationally efficient (leveraging sparse, distributed event-based information processing) means for studying embodied cognition and consciousness in robotics, addressing at the same time the problem of neurobiological explainability.

## Appendix A. Biological Plausibility and Neuromorphics

**Table A1:** Factors involved in biological plausibility [164,165,166].

Factor	Description	Impact on Biological Plausibility	Computational Approaches	Relation to SNNs and Neuromorphic Hardware
Genetics	The study of genes and their function	Determines the architecture and function of neurons and their networks	Genetic algorithms, genetic knockout models	Influences the design of neuromorphic hardware and development of SNN learning rules
Anatomy	The study of the structure and organization of living organisms	Determines the morphology and connectivity of neurons and their networks	Tracing and imaging techniques	Informs the design of neuromorphic hardware and SNN architecture
Physiology	The study of the normal function of living organisms	Determines the electrical and chemical properties of neurons and their networks	Electrophysiology, imaging techniques	Influences the design of neuromorphic hardware and development of SNN learning rules
Metabolism	The chemical processes that occur within a living organism to maintain life	Determines the energy requirements of neurons and their networks	Metabolic modeling, imaging techniques	Affects the design of energy-efficient neuromorphic hardware
Feedback	The process of information returning to its source	Enables dynamic regulation of neural activity and adaptation to changes in the environment	Feedback control theory, dynamical systems	Informs the design of neuromorphic hardware and development of SNN learning rules
Noise	Random fluctuations in the activity of neurons and their networks	Allows for exploration of possible solutions and robustness to perturbations	Stochastic modeling, noise injection	Influences the design of neuromorphic hardware and development of SNN learning rules
Spatiotemporal dynamics	The interactions between neurons and their networks over space and time	Determines the emergence of complex behaviors and cognition	Dynamic field theory, network modeling	Informs the design of neuromorphic hardware and development of SNN learning rules
Plasticity	The ability of neurons and their networks to change in response to experience	Enables learning and adaptation to changes in the environment	Spike-timing-dependent plasticity, Hebbian learning	Essential for the development of SNN learning rules and design of neuromorphic hardware
Neuromodulation	The process by which neurotransmitter release is regulated	Enables regulation of neural activity and adaptation to changes in the environment	Neurotransmitter modeling, modulation of ion channel properties	Influences the design of neuromorphic hardware and development of SNN learning rules

## Appendix B. Trade-Offs with Neuromorphic Chips

**Table A2:** List of neuromorphic chips.

Name	Institution/Developer	Advantages	Disadvantages
BrainScaleS [167]	Heidelberg University and the European Union	Low power consumption, high speed, and parallel computing enable large-scale neural network models.	Limited compatibility with conventional neural network models and limited accessibility.
SpiNNaker [168]	University of Manchester	Energy efficiency, scalability, and parallel processing make it well-suited for large-scale simulations.	Limited accuracy and limited flexibility for implementing custom neural network models.
TrueNorth [169]	IBM	High energy efficiency and parallel processing capability allow for real-time processing of large-scale neural networks.	Limited flexibility in network topology and limited scalability.
Loihi [1]	Intel	Low power consumption and support for a wide range of neural network models make it highly versatile.	Limited scalability and limited support for conventional neural network models.
DYNAPs [170]	University of Zurich	High energy efficiency, support for a wide range of spiking neural network models, and flexibility in network topology make it highly versatile.	Limited scalability and limited accessibility.
NeuroGrid [171]	Stanford University	High speed and high channel count for dense neural networks	Limited scalability and not being a chip, rather a circuit board
Braindrop [172]	University of California, Davis	Low power consumption and high processing speed enable real-time processing of large-scale neural networks.	Limited scalability and limited accessibility

## Appendix C. SNN Simulators and SNN-based Frameworks

**Table A3:** List of SNN simulators.

Name	Programming Language(s)	Advantages	Disadvantages	Software Library Features
NEST [173]	C++, Python	Fast, supports many neuron and synapse models	Steep learning curve	Connectivity, plasticity models, synaptic delays, spike-timing dependent plasticity
Brian [64]	Python	Easy to use, flexible neuron and synapse models, code generation	Limited support for parallel computation	Multiple neuron models, synaptic plasticity, STDP
NEURON [174,175]	C++, Python, hoc	Efficient, extensive model library	Steep learning curve	Built-in models, point processes, gap junctions
PyNN [176]	Python	Supports multiple backends, large user community	Limited documentation	Model specification, parallel simulation, network analysis
BindsNET [177]	Python	Built-in machine learning, easy to use	Limited support for parallel computation	Spiking neural networks for machine learning
ANNarchy [178]	C++, Python	Flexible, allows customization	Limited documentation	Spiking neural networks, machine learning models
CARLsim [179]	C++	Efficient, supports many neuron and synapse models	Limited support for large-scale networks	Model specification, synaptic plasticity, spike-timing dependent plasticity
Nengo [62]	Python	Flexible, supports many neuron and synapse models	Limited documentation	Built-in models, model specification, visualization tools
SpykeTorch [180]	Python	Deep learning integration, GPU acceleration	Limited documentation	Spiking neural networks for deep learning
GeNN [181]	C++, Python	High-performance simulation, supports multiple platforms	Limited documentation	Support for GPUs, PyGeNN [182] wrapper for Python

**Table A4:** SNN-based Simulation Frameworks.

Brain Model	Description	Features	Applications	Challenges	I/O
Neural Engineering Framework [70,183]	Framework for building large-scale neural models using SNNs and mathematical tools	Integration of sensory modalities, complex behaviors	Vision, motor control, language, decision-making	Complex computations, large computational resources	Various input and output signals
Semantic Pointer Architecture [71]	Cognitive architecture using SNNs to represent and manipulate high-dimensional semantic spaces	High-dimensional representations, simulation of cognitive processes	Language, reasoning, cognitive control	Complex computations, large computational resources	Symbolic or continuous input and output signals
Liquid State Machine [184]	SNN-based model using a random network of spiking neurons to simulate the behavior of recurrent neural networks	Dynamic and adaptable behavior, sensory processing, pattern recognition, motor control	Sensory processing, pattern recognition, motor control	Large number of neurons and connections, computationally expensive	Various input and spiking or rate-coded output signals
Dynamic Field Theory [153,154]	SNN-based model using a network of neurons arranged in a continuous field to represent spatial and temporal variables	Representation of continuous variables, spatial navigation, object recognition, action planning	Spatial navigation, object recognition, action planning	Computationally expensive computations, careful optimization required	Continuous input and output signals
Neuroconstructivism [151]	Theory of brain development that proposes that the brain is shaped by the interaction between genetic factors and the environment	Interaction between genes and environment, critical periods of development	Understanding brain development and developmental disorders	Lack of consensus on the mechanisms underlying neuroconstructivism (e.g., SNN, genetics)	Environmental stimuli input and behavioral output

## Appendix D. VSA Features

**Table A5:** VSA features and their relation to SNNs [74,76,185,186,187].

VSA Feature	Description	SNN Relation
Distributed Representation	Information encoded as pattern of activation across neurons	Can use population coding for encoding
Binding	Combine distributed representation into single representation	Synchronize neural populations through oscillatory activity
Superposition	Represent multiple patterns in a single distributed representation	Allow multiple populations to be active, resulting in combined activity pattern
Permutation Invariance	Represent same information regardless of presentation order	Use recurrent connections to maintain representations over time
Transformation Invariance	Represent same information regardless of orientation or position	Use population coding and receptive fields that are selective but invariant to position/orientation
Compositional Representation	Represent complex structures using simpler representations	Combine multiple neural representations using binding and superposition mechanisms
Error Resilience	Maintain accurate representation despite noise/errors	Can use population coding and integrate information over time for disambiguation
Adaptive Learning	Learn and adapt to new patterns or concepts	Can use synaptic plasticity mechanisms and reinforcement learning
Scalability	Scale to larger or more complex tasks or datasets	Use distributed processing and hierarchical organization
Energy Efficiency	Perform computations with low power consumption	Use spiking neurons and event-driven processing, and specialized hardware

## Appendix E. Parallelism in SNNs

**Table A6:** Summary of different parallelisms in spiking neural networks.

Parallelism	Description	Example	Relationship to SNNs
Event-driven processing	Computation is only performed when events (i.e., spikes) occur, resulting in temporal sparsity. This parallelism captures the temporal sparsity inherent in SNNs, which only generate spikes when they receive sufficient input.	A neuron only produces a spike when it receives sufficient input.	Captures the temporal sparsity inherent in SNNs
Population coding	Information is represented by the activity of a population of neurons rather than individual neurons, resulting in spatial sparsity. This parallelism captures the spatial sparsity inherent in SNNs, which use distributed populations of neurons to encode information.	A group of neurons in the visual cortex encoding the orientation of a visual stimulus.	Captures the spatial sparsity inherent in SNNs
Distributed computing	The network is divided into smaller subnetworks that can be processed independently, resulting in parallelism. This parallelism enables parallel processing in large-scale SNNs, which can be challenging to simulate on a single processor.	Dividing a large network into multiple smaller networks and processing each on a separate computing node.	Enables parallel processing in large-scale SNNs
Time-multiplexed computation	Time is divided into discrete intervals, each corresponding to a computation, resulting in efficient parallel processing. This parallelism enables efficient use of computing resources in SNNs, which require substantial computational resources to simulate.	Multiple computations are performed sequentially during each time step, allowing for efficient use of computing resources.	Enables efficient use of computing resources in SNNs

## Appendix F. Event-Based Cameras

**Table A7:** List of event-based cameras.

Name	Advantages	Disadvantages	Software Stack
Dynamic Vision Sensor (DVS) [188]	High temporal resolution, low latency, low power consumption	Limited spatial resolution, noise, requires specialized event-based processing algorithms	jAER [189], ROS [190], DAVIS API
Asynchronous Time-based Image Sensor (ATIS) [191]	High temporal resolution, low latency, low power consumption, improved spatial resolution compared to DVS	Limited dynamic range, requires specialized event-based processing algorithms	jAER, ROS, ATIS driver
Dynamic and Active-pixel Vision Sensor (DAVIS) [192]	High temporal and spatial resolution, improved dynamic range and low latency compared to DVS and ATIS	Higher power consumption than DVS and ATIS	DAVIS API, jAER, ROS
Prophesee [193]	High temporal and spatial resolution, improved dynamic range, low latency, low power consumption, high dynamic range	Proprietary software and algorithms, expensive	Prophesee SDK

## Appendix G. Metrics to Compare Spike Trains

**Table A8:** Metrics for comparing spike trains [194,195].

Metric	Description	Range	Interpretation
Victor-Purpura Distance [94]	Computes the distance between two spike trains as the minimum number of events required to be added or removed to make the spike trains identical	0 to max spikes between trains	Smaller distance indicates greater similarity between the spike trains
Van Rossum Distance [95]	Computes the distance between two spike trains based on the area between the spike time kernel (exponential function) and the spike time difference histogram	0 to infinity	Smaller distance indicates greater similarity between the spike trains
ISI Distance [196]	Computes the distance between two spike trains based on the ratio of the difference in inter-spike intervals to the sum of the inter-spike intervals	0 to 1	Smaller distance indicates greater similarity between the spike trains
Spike Time Tiling Coefficient [197]	Quantifies the degree to which spike times in one spike train are tiled by spike times in another spike train	0 to 1	Larger coefficient indicates greater similarity between the spike trains

## Appendix H. GNC of Robotic Systems and SNNs

**Table A9:** Comparison of traditional robotics applications, reconfigurable robots, robotics applications with SNNs, and reconfigurable robots with SNNs.

	Traditional Robotics [198]	Reconfigurable Robots [199]	Robotics Applications with SNNs [6]	Reconfigurable Robots with SNNs [200]
Purpose	Automation of specific tasks	Versatile and adaptable robots that can change their shape and functionality to perform different tasks	Automation of specific tasks with an emphasis on adaptive and intelligent behavior	Versatile and adaptable robots that can change their shape and functionality to perform different tasks with the use of SNNs
Control	Rigid control systems, often programmed with if-then rules	Control systems that allow the robot to change its shape and functionality in response to different tasks or environments	Control systems that incorporate learning and adaptation through the use of SNNs	Control systems that allow the robot to change its shape and functionality in response to different tasks or environments and incorporate learning and adaptation through the use of SNNs
Perception	Use of sensors to detect and measure the environment	Use of sensors to detect and respond to changes in the environment and the robot’s own configuration	Use of sensors with SNNs to process sensory information and extract relevant features	Use of sensors to detect and respond to changes in the environment and the robot’s own configuration, and use of SNNs to process sensory information and extract relevant features
Behavior	Predefined behavior that is executed based on input from sensors and control systems	Behaviour that is adapted to different tasks and environments through reconfiguration of the robot’s body	Behaviour that is learned and adapted based on input from sensors and SNNs	Behaviour that is adapted to different tasks and environments through reconfiguration of the robot’s body and learned and adapted based on input from sensors and SNNs
Learning	Limited or no learning capabilities	Learning through trial-and-error and optimization of the robot’s shape and functionality for different tasks and environments	Incorporation of supervised, unsupervised, and reinforcement learning methods to improve performance and adapt to changing environments	Learning through trial-and-error and optimization of the robot’s shape and functionality for different tasks and environments, and incorporation of supervised, unsupervised, and reinforcement learning methods to improve performance and adapt to changing environments
Benefits	Improved efficiency and accuracy in completing tasks	Increased adaptability and versatility in changing tasks and environments	Improved adaptability and intelligence in completing tasks	Increased adaptability and intelligence in completing tasks
Limitations	Limited ability to adapt to new or changing environments and tasks	Complexity of design and control, and limitations in hardware and sensor technology	More flexible and adaptable, but may require significant computational resources and training time	Complexity of design and control, and limitations in hardware and sensor technology, and may require significant computational resources and training time
